# Effect of household bleach on the structure of the sporocyst wall of *Toxoplasma gondii*

**DOI:** 10.1051/parasite/2021066

**Published:** 2021-10-07

**Authors:** Aurélien Dumètre, Jitender P. Dubey, David J.P. Ferguson

**Affiliations:** 1 Aix Marseille Univ, IRD, AP-HM, SSA, VITROME 13005 Marseille France; 2 IHU-Méditerranée Infection 13005 Marseille France; 3 United States Department of Agriculture, Agricultural Research Service, Beltsville Agricultural Research Center, Animal Parasitic Diseases Laboratory Building 1001 Beltsville 20705-2350 MD United States; 4 Department of Biological and Medical Sciences, Faculty of Health and Life Science, Oxford Brookes University OX3 0FL Oxford United Kingdom; 5 Nuffield Department of Clinical Laboratory Science, University of Oxford, John Radcliffe Hospital OX3 9DU Oxford United Kingdom

**Keywords:** *Toxoplasma gondii*, Oocyst, Sporocyst, Wall, Bleach, Resistance

## Abstract

*Toxoplasma gondii* oocysts are responsible for food- and water-borne infections in humans worldwide. They are resistant to common chemical disinfectants, including chlorinated products, presumably due to the structure and molecular nature of the oocyst wall but also the sporocyst wall. In this study, we used fluorescence microscopy and transmission electron microscopy to characterise the structure of both the oocyst and sporocyst walls, exposed to household bleach. Bleach removed the outer layer of the oocyst wall and the outer layer of the wall of sporocysts exposed due to rupture of the oocyst wall. The loss of the outer sporocyst wall layer was associated with a decrease in its autofluorescence, which can be linked to the degradation of dityrosine cross-link proteins, and loss of *Maclura pomifera* lectin-reactive glycoproteins. This study suggests that the inner layers of the oocyst and sporocyst walls are the main structures responsible for the resistance of the parasite to household bleach.

## Abstract (fr)

Les oocystes de *Toxoplasma gondii* sont responsables chez l’homme d’infections cosmopolites d’origine alimentaire et hydrique. Ils sont résistants aux désinfectants chimiques usuels, notamment aux produits chlorés, vraisemblablement en raison de la structure et de la nature moléculaire de la paroi de l’oocyste mais aussi de celle du sporocyste. Dans cette étude, nous avons utilisé la microscopie à fluorescence et la microscopie électronique à transmission pour caractériser la structure de la paroi des oocystes et des sporocystes exposés à l’eau de Javel à usage domestique. L’eau de Javel élimine la couche externe de la paroi de l’oocyste et la couche externe de la paroi des sporocystes exposés en raison de la rupture de la paroi de l’oocyste. La perte de la couche externe de la paroi du sporocyste est associée à une diminution de son autofluorescence, qui peut être liée à la dégradation de polymères protéiques de dityrosine, et à une perte des glycoprotéines réactives à la lectine *Maclura pomifera*. Cette étude suggère que les couches internes des parois de l’oocyste et du sporocyste sont les principales structures responsables de la résistance du parasite à l’eau de Javel à usage domestique.

## Introduction

*Toxoplasma gondii* is a common protozoan parasite of birds and mammals worldwide. It is estimated that nearly one third of the global human population is infected by the parasite. Humans generally become infected by either ingesting tissue cysts in undercooked meat or oocysts present in water and food contaminated with cat faeces. Infections may range from asymptomatic to severe ocular, cerebral or multivisceral symptoms depending on parasite and host factors [17]. Compared to immunocompetent adults, congenitally infected children and immunocompromised people are particularly at risk of clinical toxoplasmosis. In the absence of specific drug treatments and human vaccines, preventing the transmission of the parasite is essential to reduce the burden of the infection [18].

Oocysts are resistant to various environmental conditions and inactivation procedures [21]. They can remain infective for weeks in surface water and moist soils at 4–25° C and on salads or berries stored at 2–8° C. Though oocysts can be killed in a few minutes above 60° C, they withstand common household products such as bleach and detergents, and most chemical disinfectants used by the drinking water industry such as chlorine dioxide and ozone. Recent studies indicate that the bilayered oocyst and sporocyst walls act as robust and almost hermetic barriers to protect the sporozoites from the deleterious effect of chemical disinfectants, in particular chlorinated products [9]. However, the structural and molecular basis for this resistance is not fully understood. Here, we used fluorescence and transmission electron microscopy (TEM) techniques to gain further insights on the structure of *T. gondii* oocyst walls, especially at the sporocyst level, exposed to household bleach.

## Materials and methods

### Oocyst production

Oocysts of the strains ME 49 (type II) or TgCgCa2 (atypical) of *T. gondii* were used throughout the experiments. They were harvested from faeces of cats 6–8 days after feeding infected mouse tissues as described in [6]. Oocysts were collected by flotation at 4° C from cat faeces on a 1.15 specific gravity sucrose solution, washed in distilled water and then resuspended in 10 mL of an aqueous solution containing 2% H_2_SO_4_. They were then allowed to sporulate at room temperature (RT 20–22° C) for 7 days under adequate aeration and gentle continuous shaking. Oocysts were stored in a 2% H_2_SO_4_ aqueous solution at 4° C until use. For experiments, oocysts were washed twice in distilled water and once in PBS at 5000 *g* for 5 min to remove sulphuric acid.

### Bleach and/or heat treatment

Oocysts in 100 μL PBS were sonicated in an Elmasonic S30H water bath (Elma, Singen, Germany) in a plastic 1.5-mL microtube for 7 min at RT. Under these conditions, sonication resulted in a mixture of microscopically intact oocysts and free intact sporocysts. Individual suspensions were then exposed to bleach or heat, or bleach and heat. For bleach treatment, parasites were incubated in 1 mL of a diluted household bleach solution (Lacroix Javel, Colgate-Palmolive, Bois-Colombes, France) containing 3% sodium hypochlorite in PBS for 30 min at 4° C. Parasites were then washed three times in PBS to remove bleach before further experiments. For heat treatment, either normal or bleach-treated parasite suspensions were suspended in 500 μL PBS and incubated in a dry block heater for 10 min at 80° C. Then, parasite suspensions were cooled at RT for 20 min prior to being stored at 4° C until further experiments.

### Transmission electron microscopy

To evaluate the effects of bleach and/or heat treatments on the ultrastructure of oocyst and sporocyst walls, suspensions containing either untreated or treated parasites were fixed in 2.5% glutaraldehyde in 0.1 M PBS buffer and processed for routine electron microscopy [6]. In summary, the samples were post-fixed in osmium tetroxide, dehydrated in ethanol, treated with propylene oxide and embedded in Spurr’s epoxy resin. Thin sections were cut and stained with uranyl acetate and lead citrate prior to examination in a Jeol 1200EX electron microscope.

### Autofluorescence and lectin labelling of the sporocyst wall

The autofluorescence (AF) intensity under UV excitation and lectin-binding pattern of the sporocyst wall were evaluated as an indirect approach to evaluate the effect of bleach and/or heat on the wall structure and molecules such as tyrosine-rich protein cross-links and glycoproteins. For this, untreated or treated parasite suspensions were reacted with an FITC-conjugated *Maclura pomifera* lectin (MPL) (Vector labs) at 20 μg/mL in 100 μL PBS for 90 min at 37° C as described in [3]. This lectin has a preferential specificity for *α*-linked *N*-acetylgalactosamine (GalNAc) structures and can label the sporocyst wall [3, 7]. Following three washes in PBS at 5000 ×*g* for 5 min, samples were mounted and examined on a BX51 microscope (Olympus, France) equipped with a CCD camera (XC30, Olympus), 40× and 100× objectives (Olympus), and suitable epifluorescence filters for AF (*λ*_ex_ = 330–385 nm/*λ*_em_ = 420 nm) and FITC (*λ*_ex_ = 395–475 nm/*λ*_em_ = 498 nm) detection. Bright field, AF, and FITC images of parasite suspensions over ~20 randomly selected microscopical fields per treatment condition were acquired at a constant exposure time of 200 ms as grey scale images using Cell^A^ software (Olympus, France) and analysed using ImageJ 1.46 m (https://imagej.nih.gov). AF relative intensity of the sporocyst wall was quantified in free sporocysts as described in Figure 2A. AF quantification of sporocysts within intact oocysts was not possible because of the superimposition of the sporocyst and oocyst walls. For the MPL-binding assay, sporocysts were classified as MPL-positive or MPL-negative based on the FITC fluorescence of their wall.

## Results and discussion

**Figure 1:**
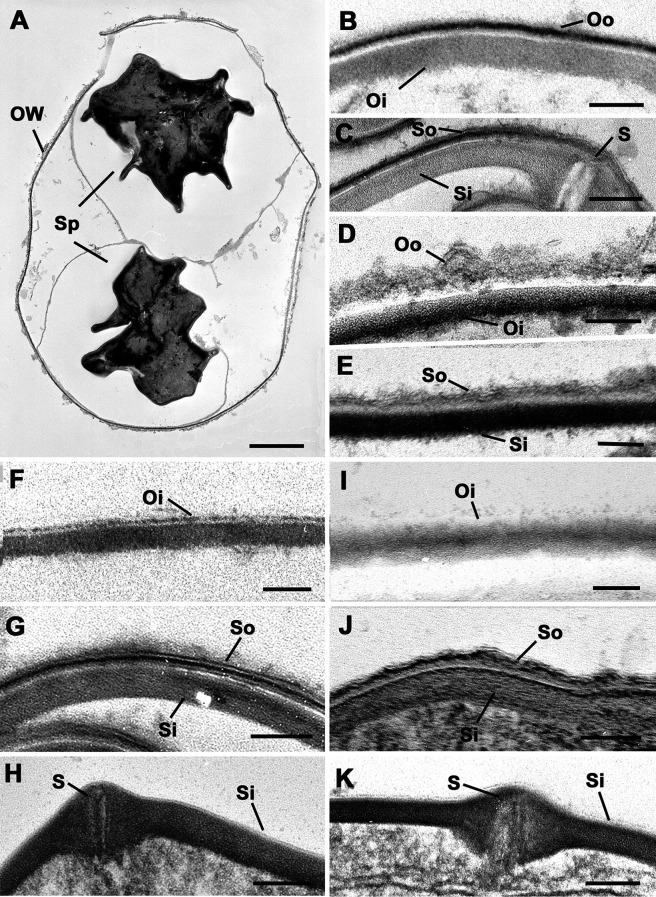
Electron micrographs of the walls of oocysts and sporocysts undergoing various treatments. A bar represents 1 μm in A and 100 nm in the other micrographs. (A) Section through an intact oocyst showing the two collapsed sporocysts (Sp). OW – oocyst wall. (B) Detail of an untreated oocyst showing the outer (Oo) and inner (Oi) layers of the oocyst wall. (C) Detail of an untreated oocyst showing the outer (So) layer and the plates of the inner (Si) layer of the sporocyst wall connected by a thicken suture (S). (D) Enlargement of a heat-treated oocyst showing the retention of both the outer (Oo) and inner (Oi) layers oocyst wall. (E) Enlargement of a heat-treated oocyst showing the retention of both the outer (So) and inner (Si) layers of the sporocyst wall. (F) Detail of the wall of an oocyst after treatment with bleach showing the inner (Oi) layer, but loss of the outer layer of the oocyst wall. (G) Bleach-treated intact oocyst showing the retention of both the outer (So) and inner (Si) layer of the sporocyst wall. (H) Naked sporocyst treated with bleach showing the loss of the outer layer, but retention of the inner (Si) layer of the sporocyst wall. S – suture. (I) Detail of an oocyst treated with bleach and heating showing the inner (Oi) layer, but loss of the outer layer of the oocyst wall. (J) Bleach and heat treated intact oocyst showing the retention of both the outer (So) and inner (Si) layer of the sporocyst wall. (K) Naked sporocyst treated with bleach and heating showing the loss of the outer layer, but retention of the inner (Si) layer of the sporocyst wall. S – suture.

**Figure 2:**
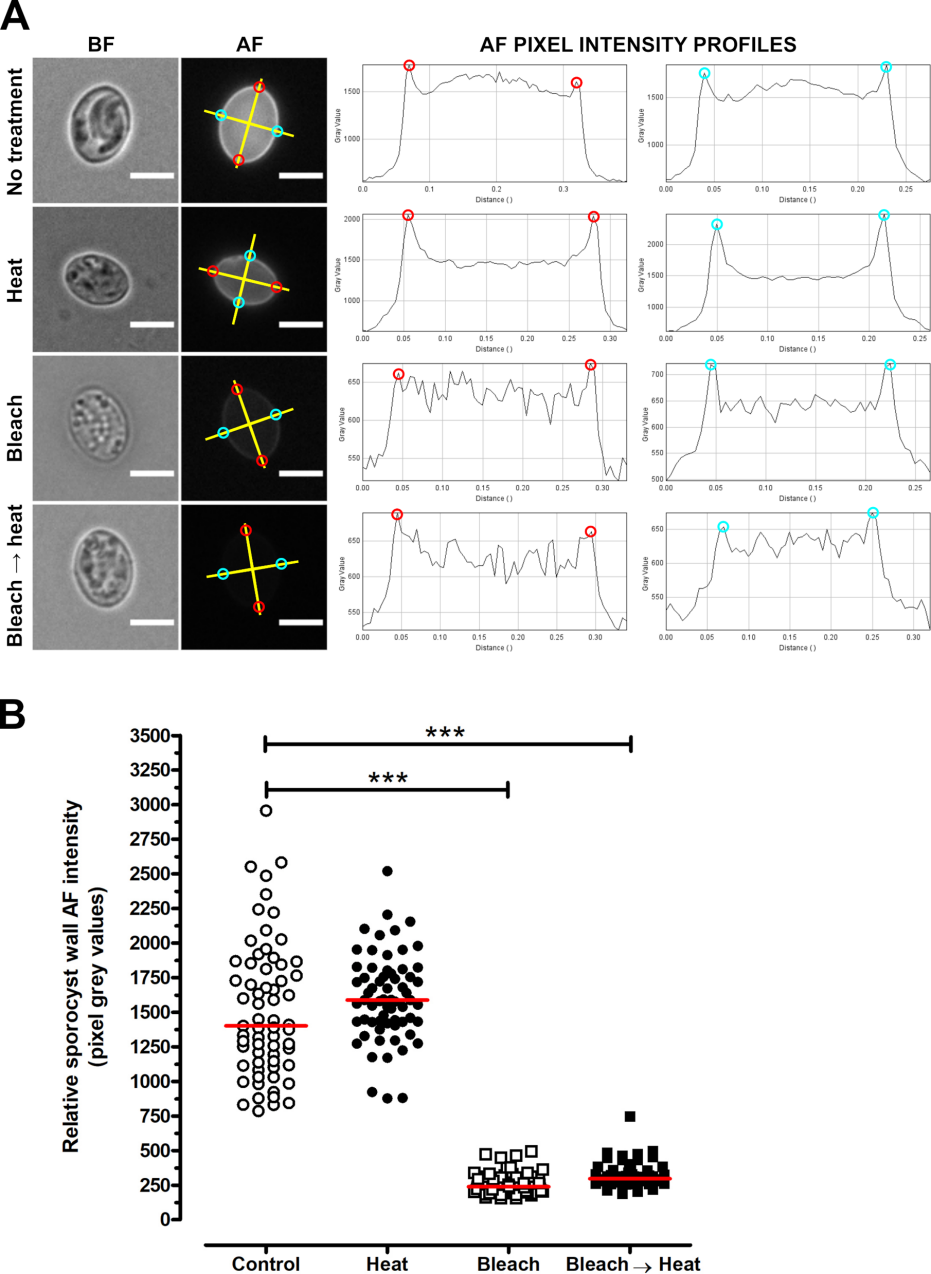
Autofluorescence (AF) wall properties of untreated, bleached and/or heated free *Toxoplasma gondii* sporocysts. (A) Relative AF intensity was quantified by recording pixel grey values along two straight lines (in yellow) arbitrarily set up across the length and width of each sporocyst. The corresponding plot profiles were generated on ImageJ to determine pixel grey values at the intercepts with the sporocyst wall (red and cyan circles). Following normalisation with regard to background grey value of each image, a mean pixel grey value was calculated for each sporocyst and then plotted as a function of parasite treatment condition using Graph Pad Prism 5.03 software. (B) Distribution of the relative AF intensity of the sporocyst wall as a function of treatment. The red line is the median of the distribution (*n* = 65 sporocysts analysed per condition). ***p < 0.001 (Kruskal–Wallis test).

**Figure 3:**
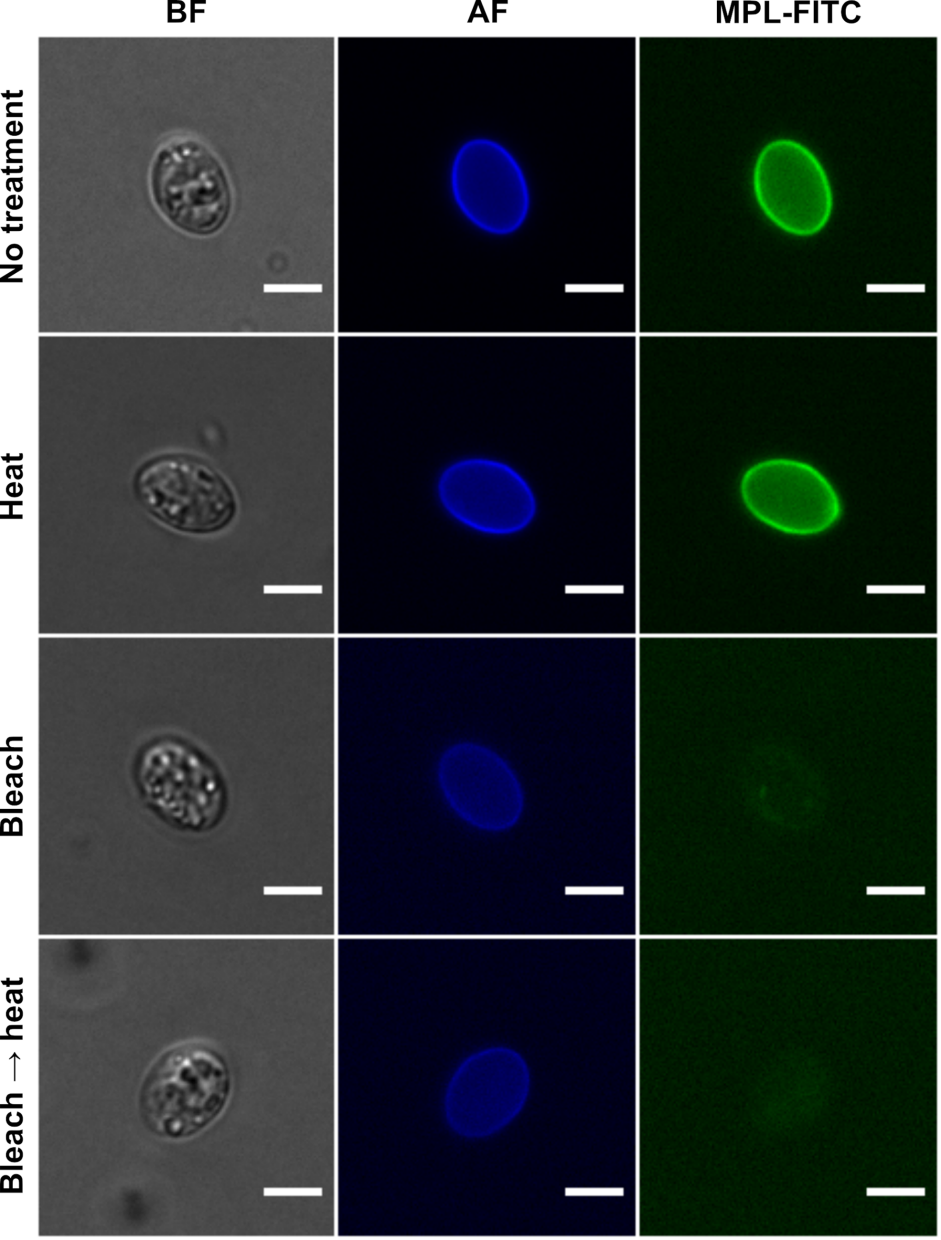
*Maclura pomifera* lectin-binding pattern of the wall of untreated, bleached and/or heated free *Toxoplasma gondii* sporocysts. BF, bright field; AF, autofluorescence; MPL-FITC, *Maclura pomifera* coupled to FITC. Bar represents 5 μm.

TEM experiments revealed that, in intact oocysts, penetration of fixatives is prevented, resulting in collapse of the sporocysts (Fig. 1A). The typical bilayered structure of the walls of untreated *T. gondii* oocysts (Fig. 1B) and sporocysts (Fig. 1C) consists of a thin outer layer and a thicker inner one. The sporocyst wall has a continuous thin outer electron dense layer, while the inner layer consists of four plates connected to each other by thickened sutures (Fig. 1C). The walls of sporocysts, whether protected or not by the oocyst wall, were similar in structure. Following heating at 80° C, both the oocyst (Fig. 1D) and sporocyst (Fig. 1E) walls retained their bilayered structure. However, after exposure to household bleach, the outer layer of the oocyst wall was stripped off, but not the inner one (Fig. 1F). Sporocysts found in intact oocysts retained their bilayered wall (Fig. 1G). In contrast, bleach removed the outer wall layer of free sporocysts, but had no marked effect on the structure of the inner layer and sutures (Fig. 1H). Similar observations were made in bleach then heat-treated oocysts and sporocysts (Figs. 1I–1K). The effects of treatment with household bleach were then specifically evaluated on the molecular properties of the wall of free sporocysts. AF intensity of free sporocysts significantly decreased following bleach, but not heat treatment (Figs. 2A and 2B). This reduction was concomitant with the loss of MPL labelling of the sporocyst wall surface (Fig. 3). Combined with the TEM observations, these results indicate that AF reduction and degradation of MPL-reactive glycoproteins of the wall of free sporocysts following bleach treatment appear to be linked to the removal of the outer sporocyst wall layer.

The exact nature, location, and functions of the molecules composing the oocyst and sporocyst walls of *T. gondii* and related coccidian parasites remain largely unknown (reviewed in [2, 9, 15]). A general model indicates that both walls are highly proteinaceous with a lipid coating, while sugar polymers are restricted to the inner oocyst wall layer. A common feature across coccidian oocysts is the AF of their walls under UV excitation [13]. In *Eimeria maxima* and *E. tenella* oocysts, tyrosine-rich proteins are associated with the development of the inner layer of the oocyst wall [1, 2, 8, 15] and of the sporocyst wall [22]. These proteins are prone to form dityrosine bonds that make the oocyst and sporocyst walls autofluorescent. Bleach can strip the outer layer of the *Eimeria* oocyst wall, but does not markedly affect the structure and AF of the inner oocyst wall layer nor of the wall of encased sporocysts [1]. Tyrosine-rich proteins identified in *Eimeria* parasites have no homologues in *T. gondii* [2, 9, 15]. In this parasite, several other tyrosine-rich proteins have been detected in the oocyst and sporocyst walls [2, 9–11]. They likely contribute to AF of both walls; however, it is not clear where they are located. Our results and previous studies show that bleach strips off the *T. gondii* oocyst outer wall layer, reduces AF of the oocyst wall, and decreases the relative abundance of tyrosine-rich proteins in oocyst wall fractions analysed by mass spectrometry [6, 11]. The sporocyst wall can retain its bilayered structure and AF as long as the oocyst wall remains resistant to bleach infiltration. In contrast, openings in or removal of the oocyst wall and then bleach treatment eliminate the outer sporocyst wall layer and reduce the sporocyst wall AF. These observations suggest that, in *T. gondii*, bleach leads to the degradation of most AF-associated proteins, putatively tyrosine-rich proteins, in both the outer layers of the oocyst and sporocyst walls. It has been shown that the *T. gondii*-specific tyrosine-rich protein TyRP1, which is found in the oocyst wall but not in the sporocyst wall, is sensitive to bleach [10]. It is likely that other wall molecules contribute to the resistance of *T. gondii* and its relatives to environmental factors. In particular, the role of cysteine-rich proteins, lectin-reactive glycoproteins, and β-1,3 glucans, which have been found in the oocyst and/or sporocyst walls of *T. gondii* and/or *Eimeria* parasites remains to be explored [3, 7, 9, 12, 15].

In conclusion, bleach can strip off the outer wall layers of both *T. gondii* oocysts and free sporocysts. These modifications are associated with the reduction of autofluorescent macromolecular complexes, presumably dityrosine cross-linked, and, at the sporocyst wall level, of glycoproteins, yet to be identified. Our results confirm the role of the inner oocyst wall layer in the structural resistance of *T. gondii* oocysts to household bleach [6] and highlight the unexpected contribution of the inner sporocyst wall layer as a second protective barrier for sporozoites in case of rupture of the oocyst wall and loss of the sporocyst outer wall layer. It is not known whether fractured oocysts can survive in environmental samples; however, experimental data indicate that free sporocysts stored at 4° C in 2% H_2_SO_4_ and PBS solutions can remain infectious for 20 days [4] and 5 months [19], respectively. Interestingly, free sporocysts are a major infective stage in the life cycle of the closely related coccidian of the genus *Sarcocystis* [16]. *Sarcocystis neurona* and *T. gondii* have a similar sporocyst wall structure [14] and AF properties [13]. As suggested for *T. gondii*, this structural organisation could help *S. neurona* sporocysts withstand environmental conditions and chemical disinfectants [5], without the assistance of the oocyst wall. Future progress in the characterisation of the oocysts of *T. gondii* and its coccidian relatives should help to identify the molecules and substructures, especially at the sporocyst and oocyst wall levels, that can make these parasites so resistant to adverse environmental conditions [20].
